# Supplementary material for: Nicotinic Acid Adenine Dinucleotide Phosphate (NAADP) Induces Intracellular Ca2+ Release through the Two-Pore Channel TPC1 in Metastatic Colorectal Cancer Cells
Source: Cancers (Basel). 2019 Apr 15;11(4):542. doi: 10.3390/cancers11040542 (PMC6521149; doi:10.3390/cancers11040542)
Supplement: Supplementary file 1 [file cancers-11-00542-s001.pdf]

Supplementary Materials: Nicotinic Acid Adenine Dinucleotide Phosphate (NAADP) Induces Intracellular Ca2+ Release Through the Two-Pore Channel TPC1 in Metastatic Colorectal Cancer Cells

Pawan Faris, Giorgia Pellavio, Federica Ferulli, Francesca Di Nezza, Mudhir Shekha, Dmitry Lim, Marcello Maestri, Germano Guerra, Luigi Ambrosone, Paolo Pedrazzoli, Umberto Laforenza, Daniela Montagna and Francesco Moccia

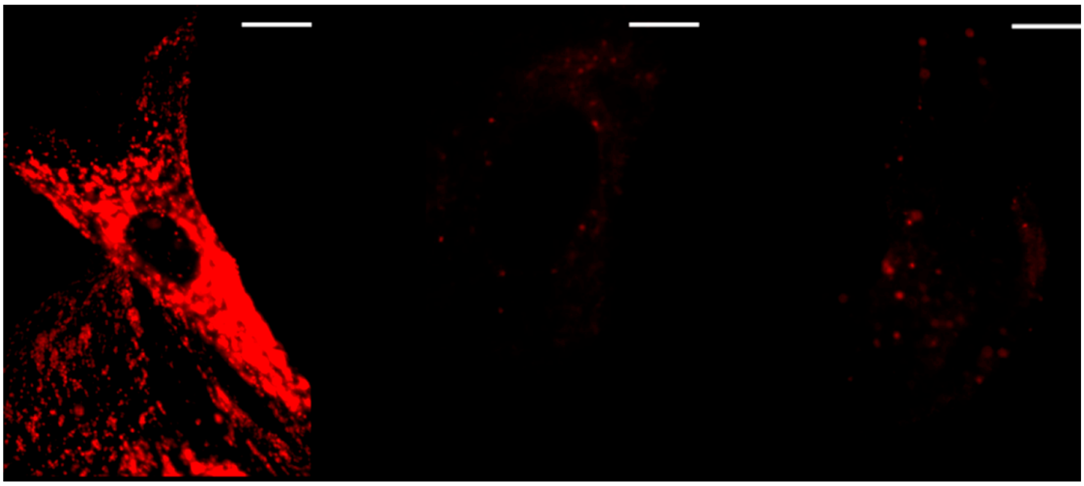

Figure S1. Disruption of acidic stores prevents Lysotracker Red staining of mCRC cells. mCRC cells loaded with LysoTracker Red (50 nM) in the absence or presence of GPN (200 μM, 30 min) or nigericin (50 μM, 30 min). The calibration bar is 5 μm.

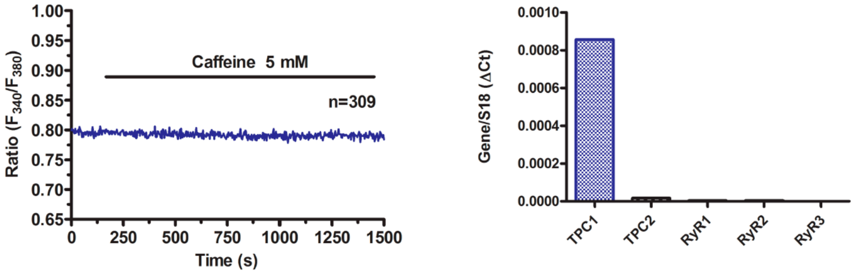

A B

Figure S2. RyRs are absent in mCRC cells. **A**, caffeine (5 mM), a selective RyR agonist, did not increase the $[Ca^{2+}]_i$ in mCRC cells. **B**, A, qRT-PCR revealed that, while TPC1 transcripts were abundantly expressed, TPC2, RyR1, RyR2 and RyR3 transcripts were undetectable in mCRC cells. Transcripts were normalized to the expression of Ribosomal Protein S18 mRNAs and expressed as mean ± SE.

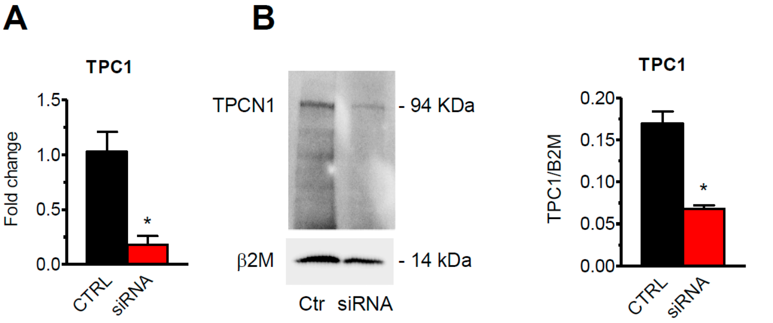

Figure S3. Genetic silencing of TPC1 through a specific siRNA in mCRC cells. Left panel, short interfering RNA (siRNA) and scrambled siRNA (CTRL) were transfected in mCRC cells as described in Materials and methods.

Blots representative of four were shown. Lanes were loaded with 30 μg of proteins, probed with affinity purified antibodies and processed as described in Materials and Methods. The same blots were stripped and re-probed with anti-beta-2-microglobulin (β2M) polyclonal antibody, as housekeeping. Major bands of the expected molecular weights were indicated. Right panel, densitometric analysis of the bands revealed a significant reduced CHRM5 protein expression in silenced cells compared to controls. The asterisk indicates $p<0.05$ (Student's t test). Densitometry was performed by Total Lab V 1.11 computer program (Amersham Biosciences Europe, Italy) and the results were normalized to the corresponding β2M.

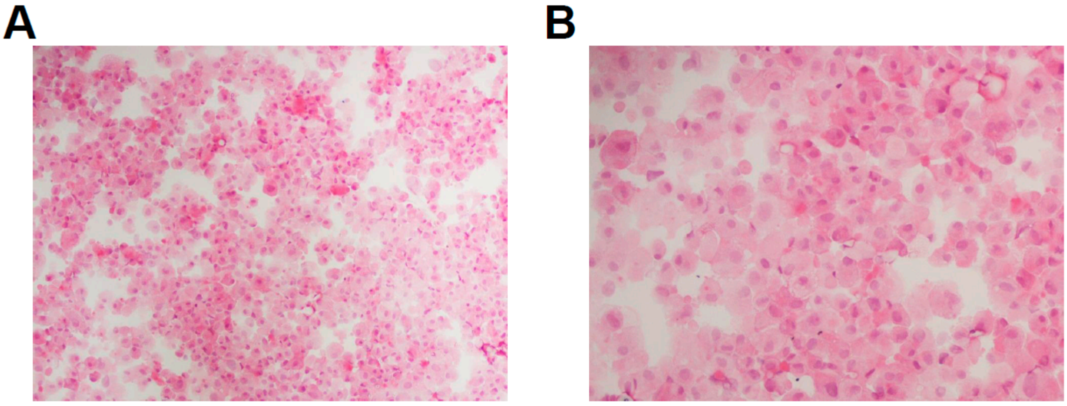

Figure S4. Immunohistochemically characterization of mCRC cells. mCRC cells were evaluated after 4–6 in vitro passages (corresponding to the timeline in which the cells were employed for molecular analysis). Cytokeratin immunofluorescence analyses of primary cultures derived from 1 patient at fifth passage (Panel A, magnification ×20, and Panel B magnification ×100) are shown.

Table S1. Primer sequences used for real time reverse transcription/polymerase chain reaction.

Gene		Primers sequences	Size (bp)	Annealing T (°C)
TPC1	Forward	5′-GAGTTTGGATGACGACGTGC-3′	132	58
	Reverse	5′-GAGTCGTGGATGGCATAGCT-3′		
TPC2	Forward	5′-CTTACCGCAGCATCCAAGTC-3′	140	58
	Reverse	5′-GTAAAGCCACATCGAGCTGG-3′		
RyR1	Forward	5′- CTTCCTGGAGCCCACTAGC-3′	115	60
	Reverse	5′-CGTGTTAGCCAGCATCTCCT-3′		
RyR2	Forward	5′- CCGCAACCATCCACAAAGAA-3′	127	60
	Reverse	5′-AAGGTGCAGATGGAGAGGTC-3′		
RyR3	Forward	5′-CGCCACCATTCATAAGGAGC-3′	158	60
	Reverse	5′-GGGCTCTGACAGATAGGGAC-3′		
S18	Forward	5′-TGCGAGGTACTCAACACCAACA-3′	108	95
	Reverse	5′-CTGCTTTCCTCAACACCACA-3′		

© 2019 by the authors. Licensee MDPI, Basel, Switzerland. This article is an open access article distributed under the terms and conditions of the Creative Commons Attribution (CC BY) license (http://creativecommons.org/licenses/by/4.0/).
